# Why Broca's Area Damage Does Not Result in Classical Broca's Aphasia

**DOI:** 10.3389/fnhum.2016.00249

**Published:** 2016-06-01

**Authors:** Alfredo Ardila, Byron Bernal, Monica Rosselli

**Affiliations:** ^1^Department of Communication Sciences and Disorders, Florida International UniversityMiami, FL, USA; ^2^Radiology Department/Brain Institute, Nicklaus's Children's HospitalMiami, FL, USA; ^3^Department of Psychology, Florida Atlantic UniversityDavie, FL, USA

**Keywords:** Broca's area, Broca aphasia, Broca complex, Broca's area aphasia, fMRI

Traditionally it has been assumed that language production is controlled by Broca's area, corresponding to Brodmann's area (BA) 44 (*pars opercularis* of the left hemisphere) (e.g., Head, 1926; Luria, 1947/1970; Goldstein, 1948; Hécaen, 1972; Damasio and Geschwind, 1984). Since about some 20 years ago, it has been considered that BA45 (*pars triangularis*) is also part of the Broca's area (Foundas et al., 1996). Some authors have referred to a more extended language production system; Hagoort (2005, 2006) proposed that there is a “Broca's complex,” including BA44, BA45, and also BA47. Lemaire et al. (2013) refer to an extended Broca's area; Kadis et al. (2016) to an expressive language network; Bernal et al. (2015) to a Broca's network; and Ardila et al. (2016) proposed a “Broca's complex” including not only left BA44 and BA45, but also BA46, BA47, partially BA6 (mainly its mesial supplementary motor area) and extending subcortically toward the basal ganglia and the thalamus.

During the last years, there has been a significant interest in re-analyzing the function of Broca's area in language (e.g., Hagoort, 2005; Thompson-Schill, 2005; Grodzinky and Amunts, 2006; Burns and Fahy, 2010). Different proposals have been presented including: binding the elements of the language (Hagoort, 2005), selecting information among competing sources (Thompson-Schill, 2005), generating/extracting action meanings (Fadiga et al., 2006); sequencing motor/expressive elements (Ardila and Bernal, 2007); cognitive control mechanism for the syntactic processing of sentences (Novick et al., 2005); construction of higher parts of the syntactic tree in speech production (Grodzinsky, 2000, 2006); and verbal working memory (Haverkort, 2005). Other authors have suggested that Broca's area subregions might be compatible with the system of prefrontal hierarchical control (Bookheimer, 2002). Koechlin and Jubault (2006) for instance, proposed that the more posterior subregions of Broca's area are preferentially engaged in language tasks based on phonological processing (in which discrete actions must be organized in time), whereas the more anterior regions including BA44, BA45, and BA47 are more precisely involved in tasks based on syntactic and semantic processing, presumably representing a higher organizational hierarchy.

Evidently, the cumulative research on the functions of Broca's area is intriguing but has not given a final answer to what is the fundamental function of Broca's area in language processing.

In the classical aphasia literature it is assumed that damage in the Broca's area is responsible for the clinical manifestations observed in Broca's aphasia (e.g., Head, 1926; Luria, 1947/1970; Goldstein, 1948; Hécaen, 1972; Damasio and Geschwind, 1984). Usually, it is assumed that Broca's aphasia includes two major impairments: apraxia of speech and agrammatism (e.g., Hécaen, 1972; Luria, 1976; Kertesz, 1979; Benson and Ardila, 1996). Only with the introduction of the CT scan did it become evident that the damage restricted to the Broca's area was not enough to produce the “classical” Broca's aphasia; extension to the insula, lower motor cortex, and subjacent subcortical and periventricular white matter is required (Alexander et al., 1990; Benson and Ardila, 1996). The mild language disturbance observed in cases of damage of Broca's areas was named “Broca's area aphasia” or “minor Broca's aphasia” or “Broca's aphasia type I (Benson and Ardila, 1996). This

type of aphasia is characterized by mildly non-fluent speech, relatively short sentences and mild agrammatism. Phonetic deviations, a few phonological paraphasias can be observed (Mohr et al., 1978) and some foreign accent can also be noticed (Ardila et al., 1988). Noteworthy, brain damage restricted to the Broca's area represents an extremely unusual clinical condition. Beginning with Broca, the overwhelming majority of Broca's aphasia patients present an extended brain damage, significantly exceeding the Broca's area (Broca, 1863; Mohr et al., 1978; Naeser and Hayward, 1978; Kertesz, 1979). Dronkers et al. (2007) using high resolution MR imaging studied the brains of the two initial cases of aphasia reported by Broca, Leborgne and Lelong, and found that both patients' lesions extended significantly into medial regions of the brain, in addition to the surface lesions described by Broca. They concluded that Broca's aphasia is associated to large lesions extending beyond the Broca's area.

From the above observations, it can be concluded that Broca's aphasia requires extensive brain lesions. Lesions restricted to Broca's area are associated with just in mild language production defects.

Using direct cortical surface recordings in neurosurgical patients it has been reported that during the cued production of words, a sequence of neural events proceeds from word memories in the temporal lobe to the articulatory movements in the motor cortical motor area. Broca's area plays a monitoring role with reciprocal interactions with temporal and frontal motor brain areas. These results corroborate that Broca's area coordinates the movement of information across extended brain circuits involved in speaking. Consequently, word production requires the participation of multiple cortical areas (Flinker et al., 2015).

Functional neuroimaging studies have demonstrated that language production not only activates Broca's area, but also a wide circuit including the surrounding areas (BA46, BA47, and the anterior insula), the supplementary motor areas, and extending subcortically (Figure 1).

**Figure 1:**
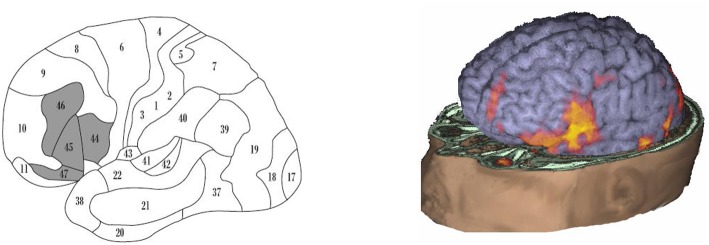
**Brain areas involved in language production**. **(Left panel)** “Broca's system” (according to Ardila et al., 2016), including BA44, BA45, BA46, BA47, mesial BA6 (supplementary motor area; not seen) and extending subcortically toward the basal ganglia and the thalamus (not seen). **(Right panel)** fMRI activation during speaking in a normal adult subject; observed is a wide activation including not only BA44 but also the surrounding areas, extending to the supplementary motor area (courtesy of the Department of Radiology− Nicklaus Children's Hospital).

If considering BA44 (and BA45) represents only a fragment of the brain system involved in language production, it becomes understandable that restricted damage in this area results in just a partial (“minor”) Broca's aphasia. During speech production, Broca's area is not the only area that becomes active, but there is an extended brain system including not only cortical but also subcortical areas. Classical Broca's area represents just a step in the brain language production system. Noteworthy, damage in other areas of this language production system (BA46, BA47, supplementary motor area, and subcortical areas—basal ganglia and thalamus) can also result in language production deficits (Papathanasiou et al., 2012), though with some idiosyncratic characteristics.

In conclusion we propose that lesions confined to the canonical Broca's area do not result in classical Broca's aphasia due to the large functional connectivity of this area with adjacent frontal and subcortical areas.

## Author contributions

AA, Primary writing; BB, fMRI analyses; MR, literature review; final writing.

### Conflict of interest statement

The authors declare that the research was conducted in the absence of any commercial or financial relationships that could be construed as a potential conflict of interest.
